# Transcriptome sequencing and metabolome analysis of food habits domestication from live prey fish to artificial diets in mandarin fish (*Siniperca chuatsi*)

**DOI:** 10.1186/s12864-021-07403-w

**Published:** 2021-02-22

**Authors:** Shan He, Jun-Jie You, Xu-Fang Liang, Zhi-Lu Zhang, Yan-Peng Zhang

**Affiliations:** 1 College of Fisheries, Chinese Perch Research Center, Huazhong Agricultural University, 1 Shizishan Street, Wuhan, 430070 Hubei China; 2 Innovation Base for Chinese Perch Breeding, Key Lab of Freshwater Animal Breeding, Ministry of Agriculture, Wuhan, 430070 China; 3 Anhui Province Key Laboratory of Aquaculture & Stock Enhancement, Fishery Institute of Anhui Academy of Agricultural Sciences, Hefei, 230031 China

**Keywords:** Mandarin fish, Transcriptome sequencing, Metabolome, H3K27 tri-methylation, DNA methylation, Food habits domestication

## Abstract

**Background:**

As economical traits, food habits domestication can reduce production cost in aquaculture. However, the molecular mechanism underlying food habits domestication has remained elusive. Mandarin fish (*Siniperca chuatsi*) only feed on live prey fish and refuse artificial diets. In the present study, we domesticated mandarin fish to feed on artificial diets. The two groups were obtained, the fish did not eat artificial diets or ate artificial diets during all of the three domestication processes, named Group W or X, respectively.

**Results:**

Using transcriptome and metabolome analysis, we investigated the differentially expressed genes and metabolites between the two groups, and found three common pathways related to food habit domestication, including retinol metabolism, glycerolipid metabolism, and biosynthesis of unsaturated fatty acids pathways. Furthermore, the western blotting and bisulfite sequencing PCR analysis were performed. The gene expression of *TFIIF* and histone methyltransferase *ezh1* were significantly increased and decreased in the fish of Group X, respectively. The total DNA methylation levels of *TFIIF* gene and tri-methylation of histone H3 at lysine 27 (H3K27me3) were significantly higher and lower in the fish of Group X, respectively.

**Conclusion:**

It was speculated that mandarin fish which could feed on artificial diets, might be attributed to the lower expression of *ezh1*, resulting in the decreased level of H3K27me3 and increased level of DNA methylation of *TFIIF* gene. The high expression of *TFIIF* gene might up-regulate the expression of genes in retinol metabolism, glycerolipid metabolism and glycerophosphoric metabolism pathways. Our study indicated the relationship between the methylation of DNA and histone and food habits domestication, which might be a novel molecular mechanism of food habits domestication in animals.

## Background

Food habits domestication can reduce production cost in animals. Mandarin fish, as an economic species, has very unique food preference. In the wild, as soon as they start to feed, they feed exclusively on live fry of other fish species [1]. Our previous study showed transcriptome determining of food preference (dead prey fish), and indicated that retinal photosensitivity, appetite control, circadian rhythm, learning and memory outputs might be involved in the food habit domestication of dead prey fish [2]. Compared to dead prey fish, the domestication of mandarin fish to accept artificial diets can provide more profitability. However, little studies investigate the molecular regulatory mechanisms of the domestication to accept artificial diets in mandarin fish.

Previous research showed that the hormones from central nervous systems play important roles in the food intake control, such as neuropeptide Y (*NPY*) and agouti-related protein (*AgRP*) [3, 4]. In giant panda, *Tas1r1* pseudogenization reinforced the herbivorous life style because of the diminished attraction of returning to meat eating in the absence of *Tas1r1* [5, 6]. The ion channels polycystic kidney disease 1-like 3 (PKD1L3) and PKD2L1 linked to sour taste, and the integral membrane protein CD36 is a putative “fat taste” receptor [7]. In a leaf-eating colobine monkey, metabolism genes *pancreatic ribonuclease* gene (*RNASE1*) was contributed to its food habits (leaves) [8]. However, little is known about the genetic and metabolic regulation on the food habits domestication of mandarin fish.

It has been noted that epigenetic status might be modified by environment and diets [9]. In mice, by feeding the diets with high levels of methyl donors (e.g. folic acid) to pregnant dams, it was possible to modify the expression of the agouti gene in the offspring with the high levels of DNA methylation [10, 11]. Histone modifications correlate with transcriptional activation and repression. The maternal undernutrition led to a decreased H3K27me3 level of the promotor region and increased expression of *pomc* gene in offspring mice [12]. Therefore, whether epigenetic regulation plays an important role in the food habits domestication is unknown.

In the present study, we domesticated the mandarin fish to accept artificial diets, and conducted the transcriptome sequencing and metabolome analysis to search the common pathways of transcriptome sequencing and metabolome. In addition, using western blotting and bisulfite sequencing PCR, we examined the methylation of histone and DNA, to investigate the molecular mechanism of food habits domestication in mandarin fish, which could promote the culture of mandarin fish with artificial diets.

## Results

### Pathway classification map of the differentially expressed genes based on transcriptome sequencing

**Fig. 1:**
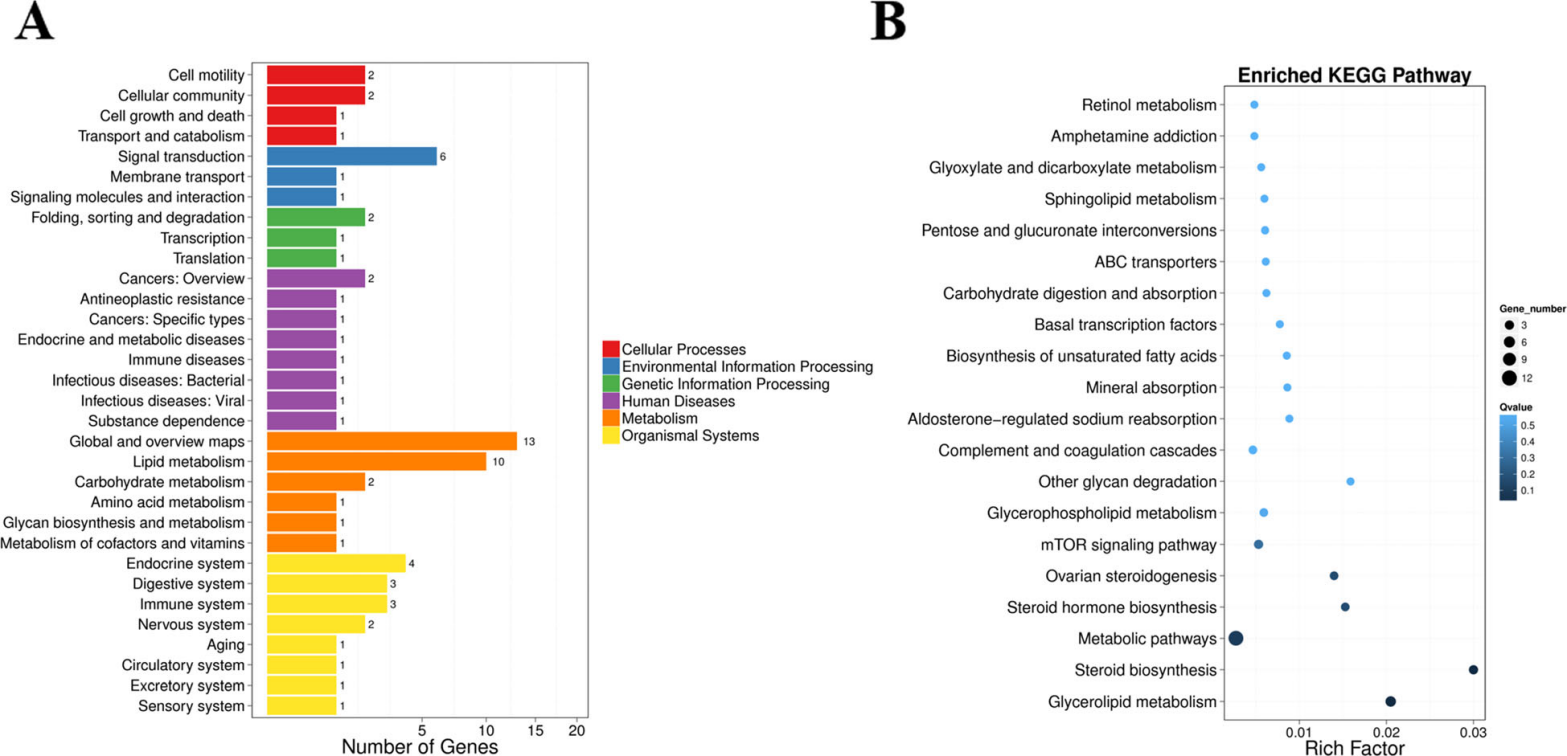
**a** Pathway classification map of the differentially expressed genes. **b** Rich factor of the differentially expressed genes of different pathway based on transcriptome sequencing

The cDNA libraries were constructed from W and X groups of mandarin fish, and sequenced using the Illumina Hiseq2000 system. High quality reads were assembled. After removing the partial overlapping sequences, a total of 77,312 distinct sequences were obtained (All-Unigene, mean size: 1138 bp, N50: 2334 bp). In these unigene, 49.06% (37,927) were less than 500 bp, 50.94% (39,385) were longer than 500 bp, in which 34.38% (26,578) were longer than 1000 bp. We found 54 genes to be differential expressed among the two groups, 29 and 25 genes are up-regulated and down-regulated in mandarin fish of Group X, respectively. The metabolic pathway showed the most differential expressed genes (Fig. 1a and b), in which lipid metabolism, signal transduction and global overview maps showed 10, 6 and 13 genes to be differentially expressed, respectively (Fig. 1a). And the rich factor of steroid biosynthesis and glycerolipid metabolism is largest of all (Fig. 1b). The details of the differential expressed genes between the two groups were presented in Table 1. The sequencing data in this study have been deposited in the Sequence Read Archive (SRA) database (accession number: PRJNA613186).

**Table 1:** Identification of differentially expressed gene based on transcriptome sequencing

Gene name	W Expression	X-Expression	log_2_FoldChange(X/W)	KEGG map	Gene function
Phosphatidate phosphatase LPIN1	43.18282	386.1148	3.160501	Glycerolipid metabolism, Glycerophospholipid metabolism, Metabolic pathways, mTOR signaling pathway.	Generating 1,2-Diacyl-sn-glycerol
3-keto-steroid reductase-like	1.127324	20.46541	4.182214	Steroid biosynthesis, Steroid hormone biosynthesis, Metabolic pathways.	Generating 4alpha-methylzymosterol or Hydroxyestrone
general transcription factor IIF subunit 2 isoform X2	1.302917	25.49286	4.290276	Basal transcription factors.	Transcription
sterol-4alpha-carboxylate 3-dehydrogenase	10.80255	140.7436	3.703626	Steroid biosynthesis, Metabolic pathways.	Generating 3-Keto-4-methylzymosterol
endothelial lipase	72.61555	753.4305	3.375124	Glycerolipid metabolism, Metabolic pathways.	Generating fatty acid
serum/glucocorticoid-regulated kinase 1	1.328233	33.50641	4.656857	mRNA surveillance pathway, cAMP signaling pathway, cGMP-PKG signaling pathway, Oocyte meiosis, Adrenergic signaling in cardiomyocytes, Vascular smooth muscle contraction, Hippo signaling pathway, Focal adhesion, Platelet activation, Long-term potentiation, Dopaminergic synapse, Inflammatory mediator regulation of TRP channels, Regulation of actin cytoskeleton, Insulin signaling pathway, Oxytocin signaling pathway.	
ATP-binding cassette, subfamily C (CFTR/MRP), member 12	1.067294	25.75915	4.593055	ABC transporters.	
stearoyl-CoA desaturase	863.601	10,415.86	3.592273	Biosynthesis of unsaturated fatty acids, Fatty acid metabolism, PPAR signaling pathway, AMPK signaling pathway, Longevity regulating pathway – worm.	Fatty acid desaturation
PH domain and leucine-rich repeat-containing protein phosphatase	4.106606	53.87121	3.713496	hospholipase D signaling pathway, Neuroactive ligand-receptor interaction, Glutamatergic synapse	
Retinol dehydrogenase 12	7.270524	99.28454	3.771438	Retinol metabolism, Metabolic pathways	Generating all-trans-Retinal
lymphokine-activated killer T-cell-originated protein kinase homolog	6.070951	145.7947	4.585872	Pancreatic secretion, Protein digestion and absorption	
5-phosphohydroxy-L-lysine phospho-lyase	1.974891	34.93728	4.144922	Lysine degradation, Metabolic pathways	Generating Allysine
transcription factor CP2-like protein 1	33.3349	1.088583	−4.93651		
serum/glucocorticoid regulated kinase 1	87.45664	1.841215	−5.56984	FoxO signaling pathway, mTOR signaling pathway, PI3K-Akt signaling pathway, Aldosterone-regulated sodium reabsorption	
hypoxia up-regulated 1	135.8593	1.88101	−6.17446	Protein processing in endoplasmic reticulum	
Alcohol dehydrogenase [NADP(+)] A	550.9179	101.9587	−2.43385	Glycolysis / Gluconeogenesis, Pentose and glucuronate interconversions, Glycerolipid metabolism, Metabolic pathways	Generating glucuronate, ethanal or D-Glyceraldehyde
Phosphatidate phosphatase LPIN1	230.0628	21.91613	−3.39196	Glycerolipid metabolism, Glycerophospholipid metabolism, Metabolic pathways, mTOR signaling pathway	
Copper-transporting ATPase 2	923.8284	210.7988	−2.13176	Mineral absorption	
calcium/calmodulin-dependent serine protein kinase	55.2038	1.86159	−4.89016	Tight junction	
solute carrier family 2, facilitated glucose transporter member 5	16.81712	0.94255	−4.15722	Carbohydrate digestion and absorption	Froctose or glucose absorption
phosphoglycolate phosphatase	15.43525	0.934372	−4.04609	Glyoxylate and dicarboxylate metabolism, Metabolic pathways, Carbon metabolism	Generating glycolate
Nuclear receptor coactivator 7	31.91563	1.559127	−4.35545	RNA degradation	
Plasma kallikrein	20,520.24	418.6751	−5.61507	Complement and coagulation cascades	
complement component 2	2119.225	73.06701	−4.85817	Complement and coagulation cascades,	

False Discovery Rate (FDR) ≤ 0.001, Fold Change ≥1.00

### Analysis of differential metabolites of two groups

We analyzed the metabolic profiles of the two groups by LC-MS in positive (ESI+) and negative (ESI−) scan modes, and selected 9249 irons for subsequent analyses (4155 irons in ESI+ mode and 5094 irons in ESI− mode).

**Fig. 2:**
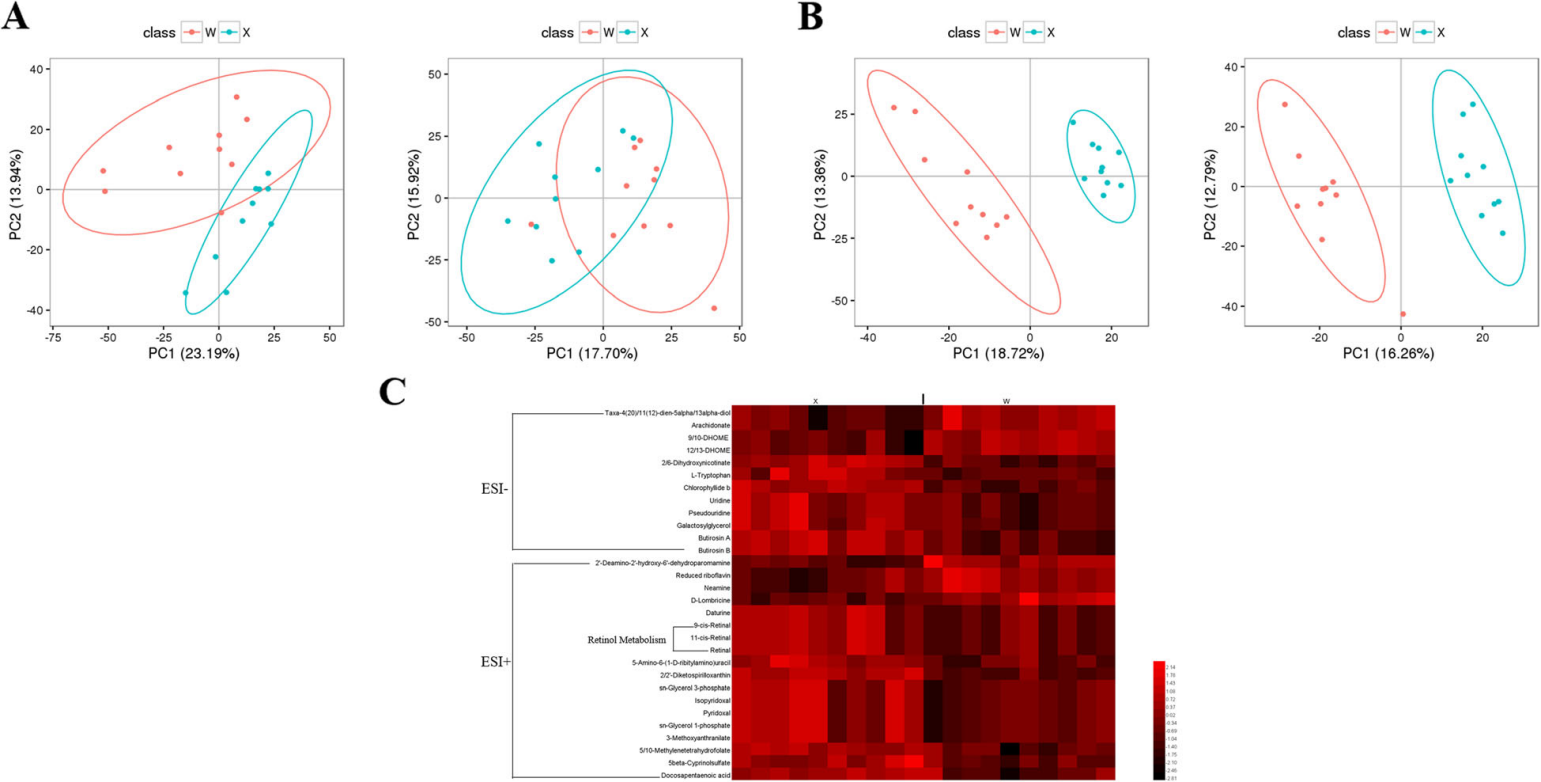
**a** PCA scores scatter plot in positive ion (left) and in negative ion (right) scan modes for the two groups. **b** PLS-DA scores scatter plot in positive ion (left) and in negative ion (right) scan modes for the two groups. **c** The heat map of differential metabolites from the related pathways between the two groups in both positive and negative mode. Each line represents a differential metabolite and each cross represents a plasma sample group. Different colors represent different abundance intensity, and the higher abundance intensity shows a gradual increase from dark color to red color

The normalized data were analyzed by PCA and PLS-DA with multivariate analysis. The PCA result showed the positive and negative ions from the different groups were in the two clusters, and were separated clearly by the first two components (Fig. 2a). PLS-DA result showed the clear separation of the two groups (Fig. 2b), suggesting the significant biochemical changes. The hierarchical clustering analysis (HCA) of the differential metabolites showed that Group X and W showed significant difference (Fig. 2c). The information of these metabolomic biomarkers was listed in Table 2.

**Table 2:** Potential metabolomics biomarkers identified between the two groups

Metabolities	RT (min)	m/z	Score	Fragfentation sscore	VIP
ESI+					
Isopyridoxal	3.577216667	190.0465891	37.7	0.344	1.41246605
Pyridoxal	3.577216667	190.0465891	37.6	0	1.41246605
sn-Glycerol 3-phosphate	3.577216667	190.0465891	36.6	0	1.41246605
5,10-Methylenetetrahydrofolate	7.691483333	475.2050752	45.1	35.4	1.93838797
5-Amino-6-(1-D-ribitylamino)uracil	7.78	259.1044545	27.4	0	1.14846268
L-Hyoscyamine	7.808933333	307.201348	38.8	5.95	1.50522082
11-cis-Retinal	7.808933333	307.201348	35.9	0	1.50522082
Littorine	7.808933333	307.201348	38.8	5.95	1.50522082
Vitamin A aldehyde	7.808933333	307.201348	35.9	0	1.50522082
2,2′-Diketospirilloxanthin	8.683883333	625.4260955	35.1	0	2.12500943
Docosapentaenoic acid	7.603166667	313.2493985	35.2	0	1.8324347
Reduced riboflavin	9.84055	361.1483341	36	0	2.03476142
sn-Glycerol 1-phosphate	3.577216667	190.0465891	36.6	0	1.41246605
2′-Deamino-2′-hydroxy-6′-dehydroparomamine	7.588333333	305.132886	34.4	0.0568	1.25836276
Neamine	9.84055	361.1483341	38.3	0	2.03476142
3-Methoxyanthranilate	3.577216667	190.0465891	38.5	4.38	1.41246605
9-cis-Retinal	7.808933333	307.201348	35.9	0	1.50522082
5beta-Cyprinolsulfate	7.897433333	550.3406162	37.1	6.39	1.88072292
D-Lombricine	5.166666667	271.0812489	37.4	0	1.77701759
ESI-					
L-Tryptophan	3.5915	203.0817932	57.9	95.1	1.62394934
Arachidonate	7.391183333	303.2325012	37.6	0	2.56862593
Uridine;	1.374833333	243.061434	56.1	88.4	2.24617127
Taxa-4 (20),11 (12)-dien-5alpha,13alpha-diol	7.391183333	303.2325012	56.1	92.4	2.56862593
Chlorophyllide b	8.206233333	627.2113855	33.4	0	2.66318502
Galactosylglycerol	1.395566667	289.0671401	37	0	2.13222525
(12Z)-9,10-Dihydroxyoctadec-12-enoic acid	7.199016667	313.2378713	43.6	31.9	2.29817224
(9Z)-12,13-Dihydroxyoctadec-9-enoic acid	7.199016667	313.2378713	43.6	31.9	2.29817224
Galactosylglycerol	1.395566667	289.0671401	37	0	2.13222525
Pseudouridine	1.374833333	243.061434	38.4	0	2.24617127
2,6-Dihydroxynicotinate	0.604766667	154.0141467	38.5	0.394	2.76092204
Butirosin B	7.66915	554.2720652	36.1	0	3.08500393
Butirosin A	7.66915	554.2720652	36.1	0	3.08500393

To identify the metabolites, we used the freely accessible database of Kyoto Encyclopedia of Genes and Genomes (KEGG) to elucidate the putative function of the metabolites. 44 and 20 irons were identified by MS1 and MS2 level in positive mode respectively, and 24 and 11 irons in MS1 and MS2 level in negative mode respectively. The details of differential ions between the two groups were presented in Table 3.

**Table 3:** Identification of differential ions based on metabolome

Differential ions					Metabolites identification					
Comparison among groups	Detect mode	Total ion number	Up-regulated	Down-regulated	MS1 ions number			MS2 ions number		
					Total	Up-regulated	Down-regulated	Total	Up-regulated	Down-regulated
X vs W	Positive	127	86	41	44	32	12	20	16	4
	Negative	116	65	51	24	17	7	11	7	4

The number of all of the differential m/z between the two groups, which including identified ions and unable identified ions

MS: the number of identified ions by searching KEGG database associated with primary data (parent ions)

MS2: the number of identified ions by searching fragmentation information available from KEGG database

### The common pathways of differential metabolites and genes

**Fig. 3:**
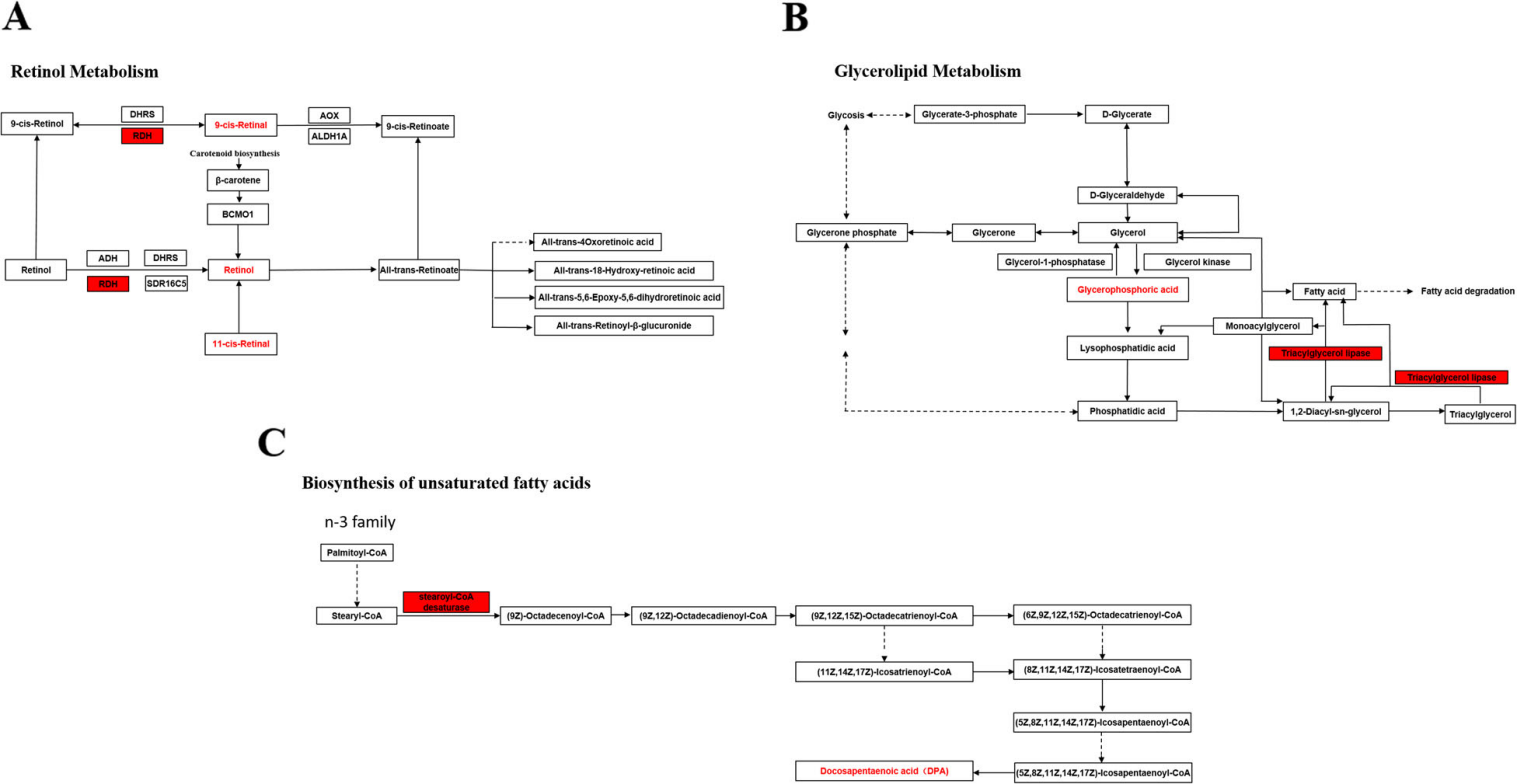
Pathways of the differentially expressed genes and metabolites based on transcriptome and metabolome. **a** Retinol metabolism; **b** Glycerolipid metabolism; **c** Biosynthesis of unsaturated fatty

In retinol metabolism pathway, retinol, 9-cis-retinol and 11-cis-retinol metabolites were higher in mandarin fish of Group X than those of Group W, RDH (retinol dehydrogenase) gene expression was consistently higher in Group X (Fig. 3a). In glycerolipid metabolism pathway, triacylglycerol lipase gene expression was higher in mandarin fish of Group X, and glycerophosphoric metabolites was also higher in Group X (Fig. 3b). In biosynthesis of unsaturated fatty acids pathway, stearoyl-CoA gene expression and DPA (docosapentaenoic acid) metabolites were higher in fish of Group X than those in Group W (Fig. 3c).

### *TFIIF* gene expression and DNA methylation

**Fig. 4:**
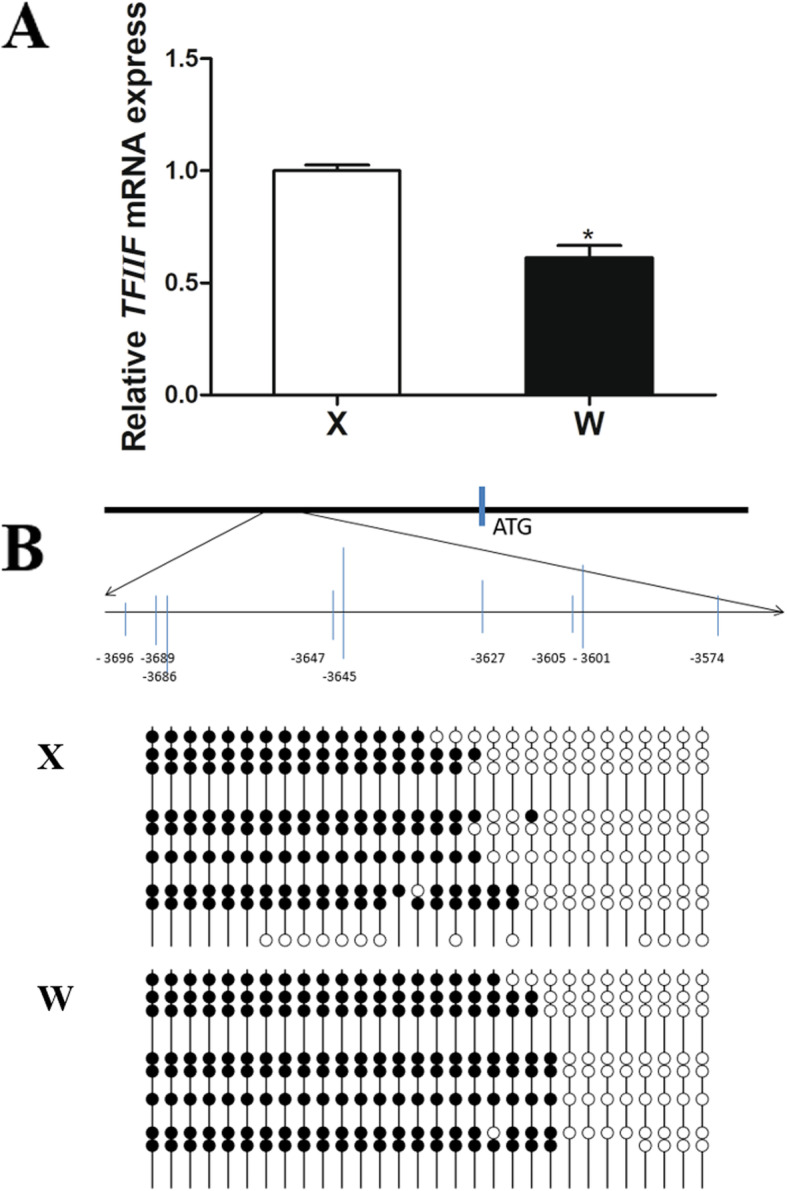
*TFIIF* gene expression and DNA methylation. **a** *TFIIF* gene expression. **b** Illustration of the region of CpG islands sites, which includes 9 CpG sites, DNA methylation patterns of the two groups (X and W) analyzed by BSP. Each line represents one individual bacterial clone, and each circle represents one single CpG dinucleotide. Open circles show unmethylated CpGs and black circles show methylated CpGs

As is shown in Fig. 4a, *General transcription factor IIF* (*TFIIF*) gene expression was higher in the mandarin fish of Group X than that of Group W. We then analyzed the CpG islands at − 5000 bp upstream from the transcription initiation site (designated as 0) of *TFIIF* by methylation analysis software. As shown in Fig. 4b, one CpG islands containing 9 CpG sites existed in − 3619 to − 3574 bp of *TFIIF* gene. The total DNA methylation level was significantly higher in the fish of Group X than that of Group W (Table 4).

**Table 4:** Methylation of each CpG island of *TFIIF* gene between the two groups

CpG Position	− 3696	− 3689	− 3686	− 3647	− 3645	−3627	− 3605	− 3601	− 3524	total
ME-CPG X	9/30 63.3%	21/30 70.0%	21/30 70.0%	22/30 73.3%	22/30 73.3%	22/30 73.3%	21/30 70.0%	22/30 84.6%	0/0 0.0%	170/236 72.0%
ME-CPG W	15/30 50.0%	18/30 60.0%	17/30 56.7%	19/30 63.3%	17/30 56.7%	18/30 60.0%	19/30 63.3%	19/29 65.5%	0/13 0.0%	142/252 56.3%
significance	0.114	0.417	0.284	0.405	0.176	0.273	0.584	0.105	\	0.000*

### *Ezh1* gene expression and histone methylation

**Fig. 5:**
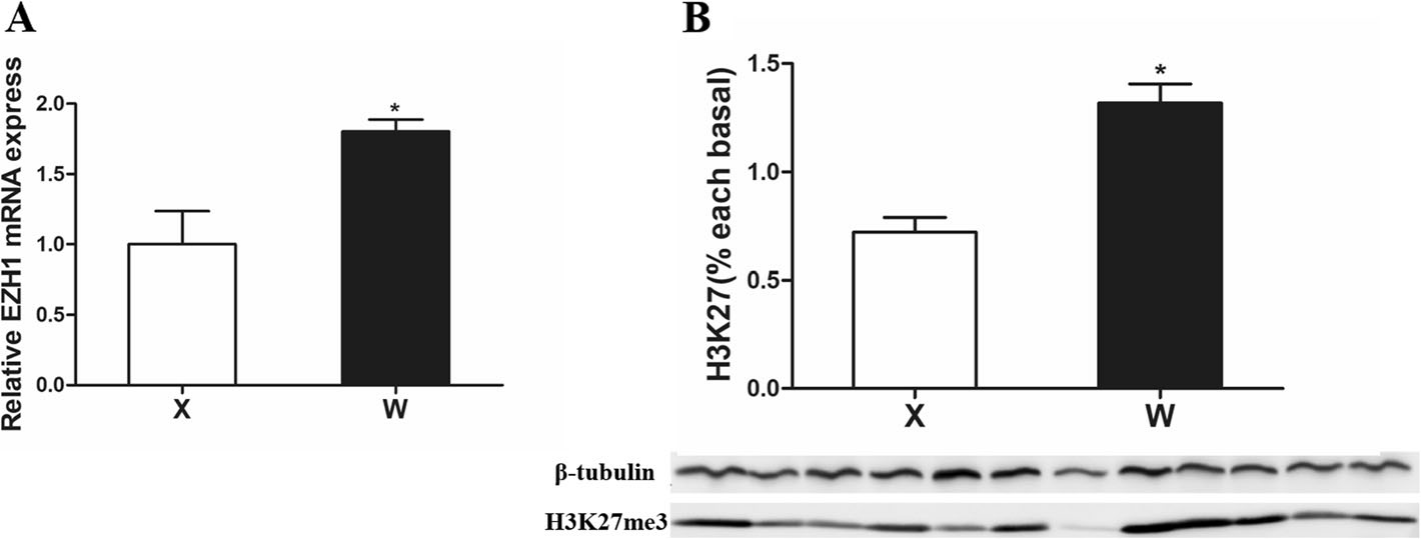
**a** Validation of *ezh1* mRNA expression. **b** The H3K27me3 protein level of between Group X and W. Data are mean ± SEM (*n* = 6), significant difference is marked with an asterisk (*P* < 0.05)

The mRNA expression of histone methyltransferase *ezh1* gene was lower in the mandarin fish of Group X (Fig. 5a). As histone methyltransferase Ezh1 could methylate ‘Lys-27’ of histone H3, we analyzed the H3K27me3 levels of the two groups. The results showed that H3K27me3 level was also lower in the mandarin fish of Group X than that of Group W (Fig. 5b).

## Discussion

In rearing conditions, mandarin fish accept only live prey fish, refusing dead prey fish or artificial diets [13]. Although pervious research showed the methods of mandarin fish domestication [14], little is known about the mechanism of food habits domestication. In the present study, we domesticated the mandarin fish to feed on artificial diets, and found a part of mandarin fish could accept artificial diets easily (Group X), but another part could not accept completely (Group W). To uncover the molecular mechanism why mandarin fish refuses artificial diets, we conducted the transcriptome sequencing and metabolome analysis. The results showed that the differentially expressed gene between the two groups were enriched in metabolism, in which the global and overview maps and lipid metabolism were the most enriched. And the rich factors of steroid biosynthesis and glycerolipid metabolism were the highest. The metabolome results showed that the pathways with different metabolites were mostly enriched in the metabolic pathways, which were consistent with transcriptome sequencing results. Previous research has shown that the most important pathways related to the domestication of dead prey fish in mandarin fish included the retinal photosensitivity, circadian rhythm, appetite control, learning and memory pathway [2]. Our results showed that metabolism, especially lipid metabolism, might contribute to the domestication of artificial diets, which was different from the domestication of dead prey fish, as the different constituents between dead prey fish and artificial diets.

We then analyzed the pathways in which the differential genes or metabolites were involved, the common pathways which showed the most enriched differential genes and metabolites, were retinol metabolism, glycerolipid metabolism and biosynthesis of unsaturated fatty acids. For retinol metabolism, retinol, 9-cis-retinol and 11-cis-retinol metabolites were higher in the Group X, consistently the *RDH* (*retinoldehydrogenase*) gene expression was higher in the Group X, suggesting a better visual acuity in the mandarin fish which could be easy to accept artificial diets. Animals make food choices on the basis of the nutritional, physiological, environmental, and sociocultural factors [7], sensory system is of significance to food choices. It is critical for mandarin fish to catch prey fish though the perception of shape and motion with well-developed scotopic vision [13]. *Salmo spp.* shows the same motion and shape of food, they have high visual acuity, thus can feed swiftly by darting, the offered food pellet can be captured immediately before it falls down to the bottom of the tank [15–17]. Because of the low visual acuity and feeding only by stalking, mandarin fish can not recognize the prey before the time when food pellet fall to the bottom of tank, thus makes it difficult to feed mandarin fish with artificial diets [13]. The retinol metabolism dysfunction might be contributed to the lower visual ability in the mandarin fish which refused artificial diets.

In glycerolipid metabolism pathway, the gene expression of triacylglycerol lipase was higher in the mandarin fish of Group X, and the glycerophosphoric acid metabolite was also higher in the Group X. In the biosynthesis of unsaturated fatty acids pathway, stearoyl-CoA gene expression and docosapentaenoic acid (DPA) metabolite were higher in the Group X. These results suggested that mandarin fish which could accept artificial diets well, might be attributed to the better capacity of glycerolipid metabolism and unsaturated fatty acids biosynthesis. Live food diets (such as zooplankton) and dry formulated diets have different fat levels and influences in European grayling [18]. Artificial diets might have more fat and energy than live prey fish, suggesting that mandarin fish which accept artificial diets could make good use of fat, while the fish which refuse artificial diets could not.

To elucidate the regulatory mechanism of up-regulated gene expression in the mandarin fish of Group X, we analyzed the differentially expressed genes based on transcriptome sequencing. The results showed *TFIIF* gene expression was significantly increased in the Group X. TFIIF communicates with a number of factors to regulate gene transcription. It has been reported that TFIIF directly binds to basal factors of TFIID, TFIIE and TFIIB [19]. TFIIF has been shown to be necessary for most, if not all, preinitiation complex formation and gene transcription [20, 21]. It suggested that in the mandarin fish which accepted artificial diets, the up-regulated transcription of genes, involved in retinol metabolism, glycerolipid metabolism and biosynthesis of unsaturated fatty acids, might be contributed to the increased *TFIIF* expression.

To uncover why *TFIIF* was upregulated in the mandarin fish of Group X, the expressions of histone methyltransferases were analyzed based on transcriptome. The expression of histone-lysine N-methyltransferase *ezh1* was significantly decreased in the Group X. Histone methyltransferases EZH1 and EZH2 catalyze the tri-methylation of H3K27, which serves as an epigenetic signal for chromatin condensation and transcriptional repression [22]. In mice, Ezh1 was required for neonatal heart regeneration after myocardial infarction and overexpression of Ezh1 promoted heart regeneration by upregulating cardiac muscle growth genes [23]. Furthermore, we observed the protein level of tri-methylation of histone H3 at lysine 27 was lower in the Group X, suggesting an active function of gene expression. The decreased abundance of histone H3K27me3 was also found in *FOXO1* (*forkhead box protein O1*) in HFD (high fat diets) fed rats, which persisted even after 8 weeks of diet reversal [24]. In addition, the total DNA methylation level of *TFIIF* was significantly higher in the mandarin fish of Group X than those of Group W. The mRNA level of *TFIIF* was higher in fish of Group X, our results showed a positive effect of DNA methylation on gene expression. In soybean, the genome-wide methylation profiles showed that hyper-methylated genes had higher gene expression [25], in ventricular septal defect patients, genome-wide DNA methylation data showed 12 hypermethylated genes had a higher gene expression [26]. In Arabidopsis, upon loss of CpG methylation, there was target-specific enrichment of H3K27me3 in heterochromatin that correlated with transcriptional reactivation, it is suggested that there was an antagonistic effect between CpG methylation and H3K27me3 [27]. It is hypothesized that the lower *ezh1* expression in the mandarin fish of Group X, could be contributed to the decreased methylation at ‘Lys-27’ of histone H3, and then up-regulating the expression and methylation of *TFIIF* gene.

## Conclusions

Our research indicated the individual differences of acceptance on artificial diets in mandarin fish and the potential molecular mechanism. The mandarin fish which feed on artificial diets, could down-regulate the *ezh1* expression, repressing the tri-methylation level of histone H3 at lysine 27, and then resulting in the increased DNA methylation and mRNA expression of *TFIIF* gene. TFIIF as an important transcription factor, might regulate the expression of genes involved in retinol metabolism, glycerolipid metabolism and glycerophosphoric metabolism, and modify the acceptance on artificial diets of mandarin fish. These results suggested the potential effect of histone methylation on food habits domestication in mandarin fish.

## Methods

### Fish domestication and sampling

Mandarin fish (69.9 ± 10.2 g) were obtained from Chinese Perch Research Center of Huazhong Agricultural University (Wuhan, Hubei Province, China) and maintained in the aquarium (12 tanks, 50 fish per tank) at constant temperature (25 ± 0.5 °C). Mandarin fish were and domesticated and fed with artificial diets (Table 5) and divided into two groups: fish did not eat artificial diets and fish ate artificial diets. The fish did not eat artificial diets during the first domestication process was then fed with live fish prey for three days, starved for two days and fed with artificial diets for one day, and then we selected the fish did not eat artificial diets during the second domestication process and repeated the domestication process for one more time. The fish ate artificial diets during the first domestication process was fed with live fish prey for one days and fed with artificial diets for three days, then we selected the fish ate artificial diets during the second domestication process and repeated the domestication process for one more time. Finally, the two groups were obtained, the fish did not eat artificial diets or ate artificial diets during all of the three domestication processes, named Group W (*n* = 56) or X (*n* = 24), respectively. Six fish were used for real-time quantitative PCR. Six fish were used for western blotting. Ten fish were used for metabolome**,** and three fish were used for transcriptome sequencing.

**Table 5:** Composition of artificial diets

Ingredients	%
White fish meal	71
Corn starch	8
Fish oil	10
Vitamin premix^1^	2
Mineral premix^2^	2
Microcrystalline cellulose	2
Carboxymethyl cellulose	2
Yeast extract powder	3

Note: 1. Vitamin premix (per kg of diet): vitamin B1 (thiamin), 30 mg; vitamin B2 (riboflavin), 60 mg; vitamin B6, 30 mg; vitamin B12, 0.22 mg; vitamin D3, 5 mg; vitamin E 160 mg; vitamin K3 50 mg; folic acid, 20 mg; biotin, 2.5 mg; pantothenic acid calcium, 100 mg; ascorbic acid (35%), 250 mg; niacinamide, 200 mg; powdered rice hulls, 999 mg

2. Mineral premix (per kg of diet): MnSO4, 10 mg; MgSO4, 10 mg; KCl, 95 mg; NaCl, 165 mg; ZnSO4, 20 mg; KI, 1 mg; CuSO4, 12.5 mg; FeSO4, 105 mg; Na2SeO3, 0.1 mg; Co, 1.5 mg

The experimental fish were anesthetized with MS-222 (200 mg/L) (Redmond, WA, USA) and sacrificed by decapitation according to the ethical guidelines of Huazhong Agricultural University. Immediately after the surgical resection, the liver tissue was frozen in liquid nitrogen and stored at − 80 °C until used. The blood was drawn from the tail vein. The plasma was obtained from whole blood sample separated with 4000 r/centrifuge for 10 min. The animal protocol was approved by the Institutional Animal Care and Use Ethics Committee of Huazhong Agricultural University (Wuhan, China) (HZAUFI-2017-015).

### RNA isolation and reverse transcription

Total RNA was extracted using Trizol reagent, and one microgram of total RNA was synthesized to complementary DNA (cDNA) by Revert Aid™ Reverse Transcriptase (TaKaRa, Tokyo, Japan). The protocols were following the manufacturer’s instructions.

### Transcriptome sequencing

For transcriptome analysis, the equal amount of total RNA was used to construct the libraries for each group (*n* = 3) using MGIEasy RNA kit (BGI, Wuhan, China). The paired-end cDNA libraries were constructed and sequenced using BGISEQ-500 system (BGI, Wuhan, China). The processes of image deconvolution, base calling, unigene assembly, annotation and expression level estimation were carried out as described by You et al. [28]. The differentially expressed genes were identified as DEGseq method described before [29], and the significance of gene expression difference was judged with Fold Change ≥2.00 and FDR (False Discovery Rate) ≤ 0.001 as the threshold. The analysis of GO function and KEGG pathway of differentially expressed genes were carried out.

### Metabolome

Serum samples (40 μl, including QC samples) were added to new Eppendorf tubes with ice-cold methanol (120 μl), vortex mixed for 1 min, placed in holding for 30 min at − 20 °C, and centrifuged at 4000 g for 20 min at 4 °C. 25 μl of supernatant and 225 μl 50% methanol were mixed. Then 20 μl of mixture from each sample were mixed as quality control samples, 60 μl of mixture was conducted as samples. All samples were stored at − 80 °C (ten biological replicates for each group).

All samples were acquired by the LC-MS system followed machine orders. Chromatographic separations were performed using an ultra performance liquid chromatography (UPLC) system (Waters, USA) and a high-resolution tandem mass spectrometer SYNAPT G2 XS QTOF (Waters, USA) was used to detect metabolites as the methods described by Huang et al. [30]. The mass spectrometry data were acquired in Centroid MSE mode. Statistical analysis was performed as previous [31]. Putative metabolites were first derived by searching the exact molecular mass data from redundant m/z peaks against the online HMDB (http://www.hmdb.ca/), METLIN (http://metlin.scripps.edu/) and KEGG (www.genome.jp/kegg/) databases.

### Real-time quantitative PCR

We searched the cDNA sequences from transcriptome data of mandarin fish, designed the primers with Primer 5.0 software (Table 6). The potential housekeeping gene according to the literature [32], *beta-actin*, *b2m*, *rpl13a*, and *hmbs* were examined, and *Rpl13a* gene was more stable and selected as the internal control. Real-time quantitative PCR was performed with MyiQ™ 2 Two-Color Real-Time PCR Detection System (Bio-Rad, Hercules, USA) as the methods described by Liang et al. [33]. The target gene expression relative to *rpl13a* expression were calculated by the optimized comparative Ct (2^-ΔΔCt^) value method [34]. Data with six biological replicates and three technical replicates were presented as mean ± S.E.M.

**Table 6:** Nucleotide sequences of the primers

Primers for real-time PCR	Sequences(5′-3′)
*RPL13A*-F	CACCCTATGACAAGAGGAAGC
*RPL13A*-R	TGTGCCAGACGCCCAAG
*EZH1*-F	AAAAGATTGAGCAGCAGACA
*EZH1*-R	GGAAGCCAAACTCCACTGTA
*TFIIF*-F	GTGCCCAAATACCTCTCTCAGC
*TFIIF*-R	TCTATACCCTCAATCACAGTCAGC
Primers for BSP amplicon	Sequences(5′-3′)
BSP *TFIIF*-F	TTTAGGGTTTTGATTTTGGTTTTTT
BSP *TFIIF*-R	ACTAAATAAACAACTCTTCATTTTAC

### DNA methylation analysis

TIANamp Genomic DNA Kit (Tiangen, Beijing, China) was used for Genomic DNA extraction, and then the DNA was treated with sodium bisulfite by EZ DNA Methylation Kit (Zymo Research, USA). The BSP primers were designed by the online MethPrimer software 14 and Primer 5.0 (Table 6). As the methods described by Cai et al. [35], PCR products were subcloned and sequenced. Six samples from the mandarin fish of Group W or X were analyzed with five technical replicates.

### Western blotting

The proteins from liver tissue were separated on 10% SDS-PAGE gel and transferred onto PVDF membrane. Tri-Methyl-Histone H3 (lys27) (C36B11) Rabbit mAb was obtained from Cell Signaling Technology (Danvers, MA). The protein level H3K27me3 were detected by western blotting with the antibody (1:1000–1:4000) according to manufacturer’s instructions. Blots were probed by second antibody labeled with IR-Dye 680 or 800 cw (1:2000–1:4000, Licor, Lincoln, NE, USA) and the membranes were visualized and quantified as the methods described by You et al. [28]. (six biological replicates for each group).

### Statistical analysis

SPSS 19.0 software was used for the statistical analyses. The normality and homogeneity of variances were tested using the Shapiro-Wilk’s test and Levene’s test, respectively. The significant differences were found using one-way analysis of variance (ANOVA), followed by Duncan’s multiple range tests and Fisher’s least significant difference post hoc test. Differences with *P* < 0.05 were considered to be significant.

## Data Availability

The sequencing data in this study have been deposited in the Sequence Read Archive (SRA) database (accession number: PRJNA613186).

## Abbreviations

EZH1: Histone H3-lysine27 N-trimethyltransferase EZH1
H3K27me3: Histone H3-lysine27 tri-methylation
TFIIF: General transcription factor IIF
NPY: Neuropeptide Y
AgRP: Agouti-related protein
Tas1r1: Taste receptor type 1 member 1
PKD1L3: Polycystic kidney disease 1-like 3
PKD2L1: Polycystic kidney disease 2-like 1
RNASE1: Pancreatic ribonuclease
B2m: Beta-2-microglobulin
Rpl13a: Ribosomal protein L13a
Hmbs: Hydroxymethylbilane synthase
QC: Quality control
PCA: Principal component analysis
LC-MS: Liquid chromatography mass spectrometry
KEGG: Kyoto Encyclopedia of Genes and Genomes
RDH: Retinol dehydrogenase
DPA: Docosapentaenoic acid
GO: Gene Ontology
FOXO1: Forkhead box protein O1
HFD: High fat diets
UPLC: Ultra performance liquid chromatography
FDR: False Discovery Rate
ANOVA: One-way analysis of variance
